# Bovine Colostrum Supplementation Modulates the Intestinal Microbial Community in Rabbits

**DOI:** 10.3390/ani13060976

**Published:** 2023-03-08

**Authors:** Stella Agradi, Paola Cremonesi, Laura Menchetti, Claudia Balzaretti, Marco Severgnini, Federica Riva, Bianca Castiglioni, Susanna Draghi, Alessia Di Giancamillo, Marta Castrica, Daniele Vigo, Silvia Clotilde Modina, Valentina Serra, Alda Quattrone, Elisa Angelucci, Grazia Pastorelli, Giulio Curone, Gabriele Brecchia

**Affiliations:** 1 Department of Veterinary Medicine, University of Milano, Via dell’Università 6, 26900 Lodi, Italy; 2 Institute of Agricultural Biology and Biotechnology (IBBA), National Research Council (CNR), U.O.S. di Lodi, Via Einstein, 26900 Lodi, Italy; 3 School of Biosciences and Veterinary Medicine, University of Camerino, Via Circonvallazione 93/95, 62024 Matelica, Italy; 4 Institute of Biomedical Technologies (ITB), National Research Councili (CNR), Via Fratelli Cervi 93, 20054 Segrate, Italy; 5 Department of Biomedical Sciences for Health, University of Milan, Via Mangiagalli 31, 20133 Milan, Italy; 6 Department of Veterinary Medicine, University of Perugia, Via San Costanzo 4, 06126 Perugia, Italy; 7 Department of Agricultural, Food and Environmental Sciences, University of Perugia, Borgo XX Giugno, 74, 06121 Perugia, Italy

**Keywords:** microbiota, caecum, nutraceutical, Firmicutes, Bacteroidetes

## Abstract

**Simple Summary:**

Recently, research has focused on the modulation of the gut microbiota because of its central role in several digestive physiological functions and its involvement in the onset of not only gastrointestinal but also systemic diseases. Supplementing rabbit diets with nutraceutical substances could be a strategy to prevent dysbiosis, strengthen the immune system, and reduce mortality during the critical weaning period. Bovine colostrum (BC) is a by-product of the dairy industry and is very rich in compounds with several biological activities. Its use as an intestinal microbiota modulator in rabbits has never been investigated. This study evaluates the effects of diet supplementation with two different percentages of BC (2.5 and 5%) on luminal and mucosa-associated microbiota and its metabolism-associated pathways in the jejunum, caecum, and colon of rabbits. Although our results showed no effect of BC on microbiota biodiversity, there were significant differences between experimental groups in the microbial composition, mainly at the level of sub-dominant components depending on the dose of supplementation. The metabolism-associated pathways have also been affected, and particularly interesting are the results on the amino acids and lactose metabolism. Overall, findings suggest that BC could be used as a supplement in rabbit feed, although its effects on productive and reproductive performances, intestinal disease resistance, and economic aspects need to be further evaluated.

**Abstract:**

BC is a nutraceutical that can modulate intestinal microbiota. This study investigates the effects of BC diet supplementation on luminal and mucosa-associated microbiota in the jejunum, caecum, and colon of rabbits. Twenty-one New Zealand White female rabbits were divided into three experimental groups (*n* = 7) receiving a commercial feed (CTRL group) and the same diet supplemented with 2.5% and 5% BC (2.5% BC and 5% BC groups, respectively), from 35 (weaning) to 90 days of age (slaughtering). At slaughter, the digestive tract was removed from each animal, then both content and mucosa-associated microbiota of jejunum, caecum, and colon were collected and analysed by Next Generation 16SrRNA Gene Sequencing. Significant differences were found in the microbial composition of the three groups (i.e., beta-diversity: *p* < 0.01), especially in the caecum and colon of the 2.5% BC group. The relative abundance analysis showed that the families most affected by the BC administration were Clostridia UCG-014, Barnesiellaceae, and Eggerthellaceae. A trend was also found for Lachnospiraceae, Akkermansiaceae, and Bacteroidaceae. A functional prediction has revealed several altered pathways in BC groups, with particular reference to amino acids and lactose metabolism. Firmicutes:Bacteroidetes ratio decreased in caecum luminal samples of the 2.5% BC group. These findings suggest that BC supplementation could positively affect the intestinal microbiota. However, further research is needed to establish the optimal administration dose.

## 1. Introduction

Rabbits are herbivores with a digestive system anatomically and physiologically evolved to obtain nutrients from low-calories and fibre-rich food thanks to the intense fermentative activity in the large intestine, particularly in the caecum [1,2]. A eubiotic condition for the digestive tract microbiota is pivotal for the maintenance of health and both productive and reproductive performances [3,4]. In rabbit breeding, weaning is a very critical period because the diet changes from milk to solid food. This induces a profound modification in the intestinal microbial population resulting in the onset of enteric diseases caused by inflammatory and infectious events [5,6] due to the impaired function of the immune system. Although these inconveniences could be reduced with the use of antibiotics, the use of these drugs has been greatly cut down over the years following the European guidelines related to antibiotic resistance.

Natural substances integrated into the diet can favour the development of beneficial gut microbial flora and, therefore, adequately stimulate the immune system not only in human beings but also in animals. In rabbits, this could contribute to the reduction of the use of antibiotics and the improvement in animal welfare as well as the profitability of the farm [7]. Moreover, rabbits are valid experimental animal models to evaluate the changes induced by the diet in the commensal intestinal bacterial population [8,9], as well as the relationships between nutrition and immunological functions [10,11,12], productive [13,14,15] and reproductive performances [16,17,18,19].

Among the natural substances which can be supplemented to the feed, bovine colostrum (BC) has recently drawn the attention of the scientific community. Colostrum is the secretion produced by the mammary glands immediately after delivery [20], which is commonly treated as a by-product of the dairy industry. The main functions of BC, besides providing essential nutritional components for the newborns’ growth, are to boost the natural defence system, regulate the immune response, equilibrate the intestinal microbiota, and improve various tissues’ growth and repair [21,22]. Defense-acting substances such as immunoglobulins, lactoferrin, lysozyme, and glycomacropeptide can have a direct action on pathogens, while other substances, such as oligosaccharides, gangliosides, and nucleosides, can act indirectly by promoting the growth of beneficial microbial flora. Bacteria like Lactobacillaceae, Bifidobacteriaceae, Lachnospiraceae, Akkermansiaceae, and Bacteroidaceae can, indeed, strengthen immune defences and modulate the physiology of the digestive system [23,24,25]. In recent years, a large number of studies have evaluated the potential role of BC in the health [26,27], energy balance, and sports performance [28] of humans. Moreover, some studies have also highlighted the beneficial effects of BC supplementation for animals such as mice [29], rats [30], horses [31], piglets [32], dogs [33,34], lambs [35], poultry [36], and calves [37].

With regards to rabbits, the effects of BC supplementation have been assessed on meat quality [38] and diarrhoea prevention at weaning [39], yet its action on the gut microbiota remains unexplored. Only recently, the bacterial microbiota composition along the gastrointestinal tract of rabbits has been characterized [40,41], and its changes after dietary supplementations have been considered [8,9]. To our knowledge, changes in the intestinal microbiota induced by BC diet supplementation in rabbits have not been evaluated yet. Moreover, only in one study mucosal and luminal gut microbiota have been compared at the *Sacculus rotundus* level [42].

In this study, we hypothesized that BC-enriched diets in rabbits can influence gut bacterial richness, diversity, and functional potential and that these modifications are dose-dependent. The purpose of this experimental trial was to discern the luminal and mucosal microbiota composition, as well as their metabolism-associated pathways, in different digestive tract sections (jejunum, caecum, and colon) of rabbits fed with diets supplemented with two percentages of BC. 

## 2. Materials and Methods

### 2.1. Animals and Samples Collection

The experimental trial was conducted in the facilities of the Department of Agricultural, Food and Environmental Sciences of the University of Perugia, Italia. 

In accordance with the European and Italian laws (EU Directive 2010/63 and Decreto Legislativo 26/2014) regarding the protection of animals used for scientific purposes, the rabbits were maintained under the supervision of a responsible veterinarian, and the experimental protocol was approved by the Ethical Committee of the Department of Veterinary Medicine of the University of Milano with the code OPBA_42_2021. All efforts were made to minimize animal discomfort and to reduce the number of experimental animals.

According to dietary treatment, 21 New Zealand White female rabbits were randomly assigned to three groups from 35 days (weaning) until 90 days of age (slaughtering). The control group (*n* = 7 animals, CTRL) was fed with a commercial feed including the following ingredients: dehydrated alfalfa meal (43.0%), wheat bran (30.0%), barley (9.5%), sunflower meal (4.6%), rice bran (4.0%), soybean meal (4.0%), calcium carbonate (2.2%), cane molasses (2.0%), soybean oil (0.4%), and salt (0.3%). The other two groups were fed with the same diet, to which, before pelleting, was added lyophilized BC at the rate of 2.5% (2.5% BC group, *n* = 7) and 5% (5% BC group, *n* = 7). The lyophilized BC was derived from high-quality colostrum from multiparous dairy cows. The basic composition and IgG concentration were evaluated before lyophilisation: total solids 22.1%, fat 4.5%, total protein 14.2%, lactose 2.9%, and IgG 3.3 g/100 mL. Table 1 shows the analytical chemical composition of the feeds. The animals used in the trial were suckled by mothers who were fed the same diet they received after weaning. These diets were previously used in another experiment [38].

Rabbits were raised in individual cages and kept in a conditioned environment with a temperature ranging between 18–20 °C, relative humidity of 60–65%, and a photoperiod of 16 h of light for the duration of the entire trial; water and feed were provided ad libitum.

Rabbits were slaughtered following the EU Regulations currently in force in an authorized slaughterhouse. The animals were first stunned by mechanical stunning and then slaughtered by jugulation. The digestive tract was removed from each animal, and the content of the different intestinal tract sections (jejunum, caecum, and colon) was collected. Specifically, regarding the colon, the anatomical region sampled was the proximal part between the *Ampulla caecalis coli* and *Fusus coli*. Five cm of each intestinal tract were dissected and longitudinally opened. The content of these tracts was collected in 15 mL sterile tubes and then stored at −80 °C until analysis. Each sample was examined individually for the determination of the luminal microbiota. Moreover, the same three collected tracts of the gastrointestinal apparatus were used for the collection of the mucosa-associated microbiota. After luminal microbiota collection, the intestinal tissue was gently rinsed with a sterile saline solution to remove any residual contents. A sterile scalpel blade was then used to scrape the luminal surface of the tissue samples (2 × 2 cm) in order to collect the mucosa-attached bacteria for mucosa-associated microbiota determination. The achieved samples were stored in sterile 1.5 mL tubes at −80 °C until analysis.

### 2.2. Microbiota Evaluation–Genomic Sequencing

#### 2.2.1. DNA Extraction

The bacterial DNA was extracted from each sample of intestinal contents by using the commercial QIAamp PowerFecal Pro DNA Kit (Qiagen, Hilden, Germany), as already described [8]. The NanoDrop ND-1000 spectrophotometer (NanoDrop Technologies, Wilmington, DE, USA) was used to verify the DNA quality and quantity; the isolated DNA was then stocked at −20 °C until use. 

#### 2.2.2. 16S Ribosomal RNA (rRNA) Gene Sequencing

Bacterial DNA was amplified and sequenced as described in Cremonesi et al., 2022 [8]. Briefly, 16S rRNA amplicons were prepared following the 16S Metagenomic Sequencing Library Preparation Protocol (Illumina, San Diego, CA, USA); libraries were pooled in equimolar proportion and then sequenced in one MiSeq (Illumina) run with 2 × 250-base paired-end reads. The dataset comprised a total of 126 samples, deriving from 3 tissues (jejunum, caecum, and colon), 2 sites (lumen and mucosa), and 3 diet groups (CTRL diet, 2.5% BC, and 5% BC). Each combination had 7 independent replicates.

#### 2.2.3. Sequence Analysis

Raw reads from each sample were subjected to a preliminary filtering pipeline that comprised the merging of the two paired reads coming from the same fragment in one single sequence by PandaSeq [43] and the trimming/filtering of low-quality bases/reads (i.e., trimming from the 3′-end stretches of bases whose Phred quality score was <3; resulting fragments having a length shorter than 75% of the initial fragment length were discarded). Filtered reads were clustered into zero-radius operational taxonomic units (zOTUs) by USEARCH (v. 11.0.667, [44]) in order to merge together reads putatively coming from the same species. Only zOTUs supported by 5 or more reads were retained. Downstream analyses (including alpha- and beta-diversity evaluations) were performed in QIIME 1.9.0 suite [45]. Taxonomic assignment of zOTUs was performed by the RDP classifier [46] against SILVA 138 database [47] using 0.5 as the confidence threshold.

#### 2.2.4. Functional Predictions

Functional predictions from the 16S rRNA-derived microbial profiles were estimated by applying PICRUSt2 (v 2.5.1, [48]) on the zOTU table of the 125 samples considered in the experiment after normalization to the least sequenced samples. Abundances were converted into copies-per-millions (CPM) by using the humann2_renorm_table utility script from HUMANn2 [49]. Lineages were associated with the MetaCyc pathways, and abundances at each level were calculated by using the script categorize_by_function.py from PICRUSt (v 1.0.0, [50]).

### 2.3. Statistical Analysis

Biodiversity analysis (alpha-diversity analysis) was performed using several metrics (i.e., Shannon’s diversity, chao1 diversity index, observed species, and Faith’s phylogenetic diversity index). A non-parametric permutation-based *t*-test (equivalent to Mann–Whitney U-test), with 999 random permutations, was employed in order to assess whether the samples belonging to one experimental class were more or less diverse than those of a different class.

Microbial profile analysis (beta-diversity analysis) was based on unweighted and weighted UniFrac distances [51] and represented by a Principal Coordinate Analysis (PCoA) aimed at reducing the complexity of the variance. The “Adonis” test function (Permutational Multivariate Analysis of Variance Using Distance Matrices using pseudo-F ratios) was used in order to define whether there was a significant difference among the experimental groups, using 999 random permutations.

Composition analysis in terms of taxa relative abundances was performed by grouping the classified zOTUs to different taxonomic levels (phylum, class, order, family, genus). A Kruskal–Wallis test was employed to assess whether there was a significant difference among levels of the experimental categories on the relative abundance of the taxa, on the predicted pathway abundances, and on Firmicutes:Bacteroidetes ratio evaluation; Dunn’s post hoc pairwise test was employed if necessary. All statistical analyses and plots were performed using Matlab (v. 2008a, Natick, MA, USA).

### 2.4. Data Availability Statement

The data presented in this study are openly available in NCBI Short Read Archive (SRA) under experiment IDs SRR22879769-SRR22879893 (BioProject ID PRJNA915237, https://www.ncbi.nlm.nih.gov/bioproject/PRJNA915237, accessed on 5 January 2023).

## 3. Results

### 3.1. Sequencing Results

The microbiota structure of the rabbits’ gastrointestinal tract was characterized by a total of 2,797,273 high-quality reads (after filtering). Sample 114 (colon tract, lumen content of a 2.5% BC-treated rabbit) had a very low number of raw reads (i.e., 825) and was therefore discarded. The average read depth per sample was 22,374 ± 9372. All the downstream analyses, then, were performed on 125 samples (excluding sample 114), and all samples were normalized to the least sequenced sample (*n* = 4856). The analysis of the rarefactions curves for both the chao1 and the observed species metrics showed that the majority of the samples had a tendency toward reaching a plateau, thus suggesting that the depth of coverage was sufficient to describe the biological diversity within the samples (Figure S1). 

### 3.2. Taxonomic Composition of Gut Microbiota along the Rabbit Gastrointestinal Tract of CTRL and 2.5 and 5% BC Groups

Overall, the microbial composition of the mucosa-derived samples was less defined, with higher percentages of bacteria unclassified at lower levels, compared to the corresponding lumen samples. The degree of microbial definition at the genus level was, anyway, low due to the relatively poor characterization of rabbit metagenomes in the taxonomy reference database (i.e., SILVA). 

**Figure 1:**
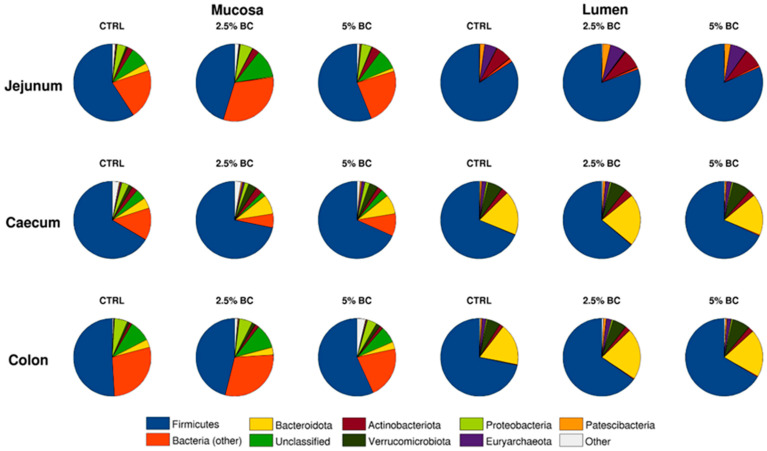
Pie charts representing the average composition of the microbiota for the samples divided by intestinal tract (jejunum, caecum, colon), sampling site (lumen, mucosa), and diet (CTRL, 2.5% BC, 5% BC) at the phylum level. Phyla with an average relative abundance <1% are reported in the “Other” category.

The major genera found in the samples were *Dubosiella*, *Akkermansia*, *Bacteroides*, *Methanobrevibacter*, *Ruminococcus*, *Marvinbryantia*, and *Alistipes*, which, however, made up only 15.5% of the relative abundance on average. Figure 1 depicts the average composition at the phylum level for the samples divided by the intestinal tract, sampling site, and diet, whereas Figure S2 summarizes the relative abundances of the samples at all taxonomic levels (phylum to genus).

Jejunum lumen samples were characterized by a relatively high content of Firmicutes (~80%), Actinobacteriota (~7%), Euryarchaeota (~6%), and Patescibacteria (~2.5%), whereas Bacteroidota were very low (<0.5%); at the family level, these samples were rich in Eubacteriaceae (~58%), Erysipelotrichaceae (~12%), Methanobacteriaceae (~6%), and Saccharimonadaceae (~2.5%).

Furthermore, the corresponding jejunum mucosa samples had about 35% of the overall abundance (on average) composed by “Unclassified” and “Bacteria” unclassified at lower levels; Firmicutes composed about 55% of the relative abundance, whereas Proteobacteria and Actinobacteriota accounted for about 4.5% and 2.5%, respectively. This was reflected also at the family level, where apart from Eubacteriaceae (accounting for ~40% of the relative abundance) and Erysipelotrichaceae (~4%), all the other families were scarcely present.

The microbiota of the caecum lumen samples was mainly composed of Firmicutes (~65%), Bacteroidota (~19%), Actinobacteriota (~2.6%), and Verrucomicrobiota (~6.5%) at the phylum level, and by Eubacteriaceae (~25%) and members of Lachnospiraceae, Oscillospiraceae, Ruminococcaceae, Akkermansiaceae, Bacteroidaceae, Christensenellaceae, as well as Muribaculaceae at the family level, each accounting for 3.9%-9.4% of the relative abundance.

Caecum mucosa samples were characterized by Firmicutes (~68%), Bacteroidota (~7%), and Verrucomicrobiota (~3%), with a higher abundance of unclassified bacteria (accounting for 7.7–18.0%), as previously observed in mucosa samples; at the family level, these samples were mainly constituted by Eubacteriaceae (~30%), Lachnospiraceae, Erysipelotrichaceae, Oscillospiraceae, and Ruminococcaceae, each in the range of 3.4–9.8% of the overall abundance.

Colon lumen samples had about ~66% Firmicutes, ~19% Bacteroidota, ~6% Verrucomicrobiota, and ~2% Euryarchaeota, whereas, at the family level, they were composed of Lachnospiraceae (9%), Ruminococcaceae (6.5%), Oscillospiraceae (6.5%), Christensenellaceae (3.5%), Akkermansiaceae (6%), Muribaculaceae (5%), and Bacteroidaceae (6%).

Finally, colon mucosa samples, besides ~50% Firmicutes, ~5% Proteobacteria, and ~3% Bacteroidota, had an additional ~35% composed of “Unclassified” bacteria; the abundance of Eubacteriaceae was the lowest (~20%), with a consistent presence of Lachnospiraceae (~8%), Oscillospiraceae (~6.5%), Ruminococcaceae (~3%), and Christensenellaceae (~2.3%).

### 3.3. Comparison of Microbiota Composition in Different Intestinal Tracts

Considering both the intestinal tract and the site (*n* = 21), jejunum mucosa samples had the lowest biodiversity (statistically different, *p* = 0.015, from all the other conditions, except jejunum lumen), followed by the jejunum lumen samples; on the other hand, caecum and colon (both lumen and mucosa) had a higher diversity (Figure S3A and Table S1). Each condition was characterized by a different microbial composition, with the beta-diversity analysis revealing significant differences on both the unweighted and the weighted UniFrac distances (*p* < 0.002 and *p* < 0.02, respectively) for all comparisons, except for caecum and colon lumen samples, which were similar (Figure S3B and Table S2). Consistent results were also found when comparing the samples over the three intestinal tracts (lumen and mucosa together, *n* = 42 per group), with the biodiversity of jejunum samples lower than the caecum and colon samples for the PD whole tree (*p* = 0.003, Figure S3C and Table S3), and the three tissues significantly separated on both unweighted (*p* = 0.001) and weighted (*p* ≤ 0.02) UniFrac distances (Figure S3D and Table S4). Similarly, this was also observed for the comparison between the lumen *versus* the mucosa samples (*n* = 63 per group), with lumen samples having a higher biodiversity than mucosa samples (*p* = 0.003, Figure S3E) and a microbial profile between the two sites significantly different, as well, on both the weighted and the unweighted UniFrac distances (*p*-value = 0.001) (Figure S3F).

### 3.4. Comparison of Microbiota Composition in Rabbits according to Bovine Colostrum Diet Supplement

**Figure 2:**
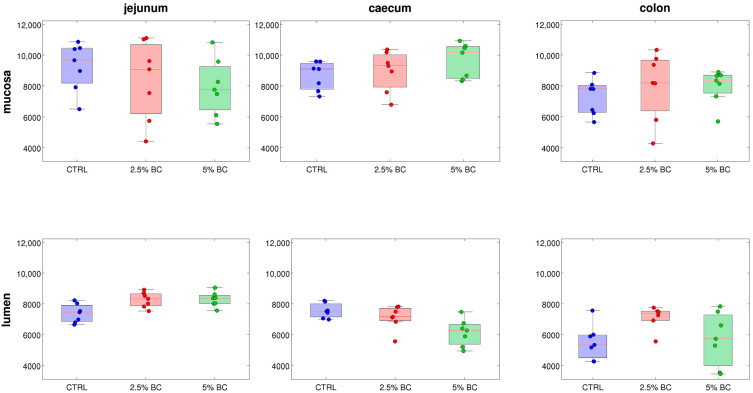
Boxplots describing the trends of chao1-based alpha-diversity for increasing % of colostrum in diets divided per intestinal tract and site. Each dot represents a sample, red lines are the median values of the distributions, the box indicates the 25% and 75% percentiles, and whiskers indicate the minimum/maximum values.

In order to determine eventual changes in microbial diversity and composition according to the BC supplementation (2.5% and 5%, as compared to the CTRL diet), a comparison of the microbiota composition in rabbits was investigated. Results were computed stratifying samples in diet groups homogeneous for the intestinal tract and site sampled (*n* = 7), i.e., considering differences only in the diet supplementation, provided that the intestinal tract (jejunum, caecum or colon) and site (lumen and mucosa) were the same. As far as the alpha-diversity analysis, there was a non-significant difference among samples according to the BC supplementation. However, according to the chao1 metric, in samples with increasing percentage of BC, the biodiversity increased in the jejunum lumen and in caecum mucosa, whereas it decreased in jejunum mucosa and caecum lumen samples; no trend was observed for colon samples (both mucosa and lumen) (Figure 2). The tendency towards increasing biodiversity in caecum mucosa was also confirmed for the observed species metric, whereas no differences were highlighted for PD whole tree and Shannon diversity indices. 

**Figure 3:**
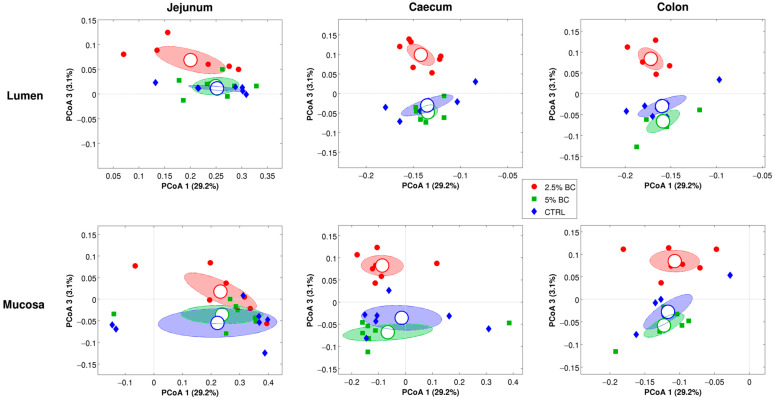
Principal Coordinate Analysis (PCoA) based on the unweighted UniFrac distances for the three diet groups divided per intestinal tract and site. In each plot, each dot represents a sample, colored according to the diet group. Ellipses are the 95% SEM-based confidence intervals, whereas centroids are the average coordinates on all the samples. Coordinates 1 and 3 are represented.

The microbial composition (beta-diversity), on unweighted UniFrac distance, of the three diet groups (CTRL, 2.5%, and 5%) resulted in significantly different (*p* ≤ 0.002 for all pairwise comparisons) in caecum and colon lumen samples. At the same time, in colon mucosa samples, significant differences were reported for CTRL vs. 5% BC and 2.5% BC vs. 5% BC, whereas no difference was observed between CTRL and 2.5% BC. No significant differences were reported for caecum mucosa and jejunum (both lumen and mucosa) samples (Figure 3 and Table S5). As a whole, the microbiota of the three diet groups (*n* = 42) did not show different biodiversity for any of the tested metrics, whereas the microbial profiles resulted somehow different for the unweighted UniFrac (*p* = 0.002, *p* ≤ 0.014) (Table S6).

**Figure 4:**
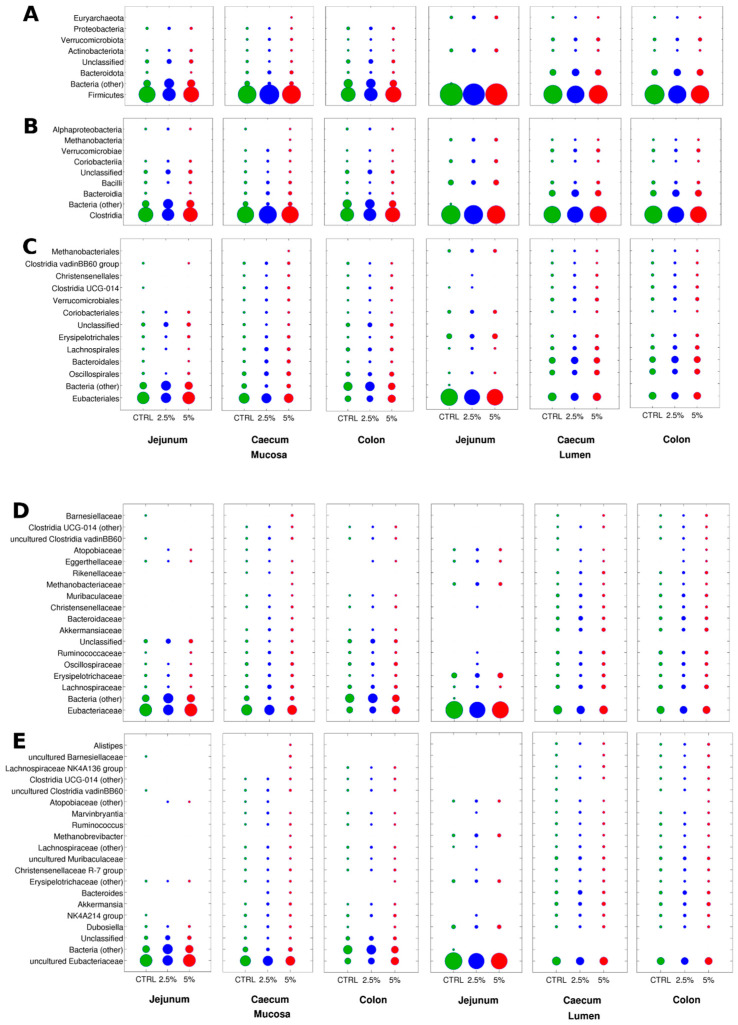
Bubble plots of the relative abundance data (for each intestinal tract and site) over the three groups at all taxonomic levels: CTRL (green), 2.5% BC (blue), and 5% BC (red); phylum (**A**), class (**B**), order (**C**), family (**D**), and genus (**E**). Circle size is proportional to the average relative abundance. For each level, only taxa with avg. rel. ab > 1% in at least one of the groups are represented. “Missing” circles are taxa < 1% in that group.

Regarding the relative abundance analysis, due to the reduced number of samples per group, the significantly altered taxa were few, and some of them were at relatively low relative abundances. This is somehow expected, also considering the results from the beta-diversity analysis, for which the differences were observed for the unweighted UniFrac distance, suggesting modifications in the sub-dominant components of the microbiota. The following data only consider the taxa present at a relative abundance > 1% on average in at least one of the experimental groups, and the indication of an “increase” or a “reduction” is provided using the CTRL diet as a reference. The reported data are for the phylogenetic level of families since it is the lowest for which the majority of the taxa are properly defined (i.e., genus-level classifications contained many “unclassified” and “uncultured” taxa). Jejunum lumen samples showed a significant increase of Clostridia in 2.5% BC and 5% BC groups, together with a tendency towards a reduction of Eubacteriaceae and Erysipelotrichaceae in the 2.5% BC group, as well as an increase of Oscillospiraceae and Ruminococcaceae in 2.5% BC group. Jejunum mucosa samples showed a tendency towards an increase of unclassified bacteria and Clostridiaceae in the 2.5% BC group, together with a tendency towards a reduction of Eubacteriaceae (in 2.5% BC) and Lachnospiraceae (in both 2.5% BC and 5% BC groups). Caecum lumen samples showed a significant increase of Erysipelotrichaceae (in 5% BC), Eggerthellaceae (in 2.5% BC and 5% BC), and family UCG-014 of Clostridia (in 2.5% BC and 5% BC), as well as a tendency towards an increase of Bacteroidaceae (in 5% BC); on the other hand, we found a significant decrease of Barnesiellaceae (in 2.5% BC) as well as of UCG-010 and vadinBB60 families of Clostridia (both in 5% BC). Caecum mucosa samples had a significant increase of Clostridia (in 2.5% BC) and a tendency towards the increase of Lachnospiraceae (in 2.5% BC), Akkermansiaceae (in 2.5% BC and 5% BC), Bacteroidaceae (in 2.5% BC), Barnesiellaceae (in 2.5% BC), and towards the reduction of Eubacteriaceae (in 5% BC) and unclassified bacteria (in 2.5% BC). Colon lumen samples were characterized by a significant increase of Eggerthellaceae (in 2.5% BC and 5% BC) and by a tendency towards an increase of Akkermansiaceae (in 5% BC), Bacteroidaceae (in 2.5% BC), and Rikenellaceae (in 5% BC); on the other hand, Ruminococcaeceae (in 2.5% BC) and Monoglobaceae (in 2.5% BC and 5% BC) were significantly reduced. Colon mucosa samples highlighted a tendency towards an increase of Eubacteriaceae (in 5% BC) and Campylobacteriaceae (in 2.5% BC and 5% BC) and a tendency towards a reduction of unclassified bacteria (in 5% BC) (Figure 4D). The complete list of altered taxa abundances is available in Table S7.

### 3.5. Functional Prediction on Microbial Profiles of Rabbits according to Bovine Colostrum Diet Supplement

The PICRUSt2-based functional prediction from the 16S rRNA microbial profiles of the samples revealed several bacterial metabolism-associated pathways significantly altered in the rabbits fed a BC-supplemented diet (Table 2). The effect of BC supplementation on predicted pathways was less evident for jejunum samples (mucosa: 13 altered pathways, lumen: 6), more consistent in the caecum (mucosa: 38 altered pathways, lumen: 10), and most abundant in the colon (mucosa: 62 altered pathways, lumen: 47). In order to better describe the metabolic functions involved, we grouped the raw pathways (“level 5”) to upper lineages (levels 1 to 4).

In the jejunum lumen, processes belonging to pathways of proteinogenic amino acid (phenylalanine and tyrosine) biosynthesis and NAD synthesis were depleted in BC groups, whereas phylloquinone biosynthesis was enriched in 2.5% BC samples; no differences were observed for 5% BC samples. On the other hand, in jejunum mucosa samples, several level-4 pathways were significantly more abundant in 2.5% BC and 5% BC, such as the biosynthesis of peptidoglycans, NAD, geranylgeranyl pyrophosphate (GGPP) (cofactor-biosynthesis group), lactose, and diterpenoids as well as isoprenoids (both methylerythritol phosphate pathways I and II were found altered), both belonging to the terpenoid-biosynthesis group and the degradation of lysine (involving L-lysine fermentation to acetate and butanoate).

In the caecum lumen, several altered pathways were reported for 2.5% BC samples and included the increase of histidine and lysine degradation and of sugar nucleotides and vitamin B6 biosynthesis (via pyridoxal 5′-phosphate biosynthesis I); at the same time, pathways related to nucleotide biosynthesis (5-aminoimidazole ribonucleotide and purine nucleotides salvage), and proteinogenic amino acid synthesis (threonine, lysine, phenylalanine, and tyrosine) were depleted. On the other hand, results for 5% BC samples were contrasting, with phenylalanine and tyrosine synthesis increased. In caecum mucosa samples, contrastingly, only one level-4 pathway was altered, i.e., histidine degradation, which resulted in more abundant samples from BC-supplemented rabbits than those fed the CTRL diet.

Colon lumen samples were characterized by a significant increase in quinone biosynthesis, including DHNA, demethylmenaquinone (pathways of demethylmenaquinol-6, -8, and -9), menaquinone (pathways of menaquinol 6–13), phylloquinone, vitamin B6 biosynthesis, and glutamate degradation in both 2.5% BC and 5% BC, whereas the biosynthesis of glycogen, acetylmuramoyl-pentapeptide, GGPP, purine nucleotides salvage, and isoprenoids was depleted. Finally, colon mucosa samples showed a significant increase in histidine and lysine degradation, and many level-4 pathways were depleted in BC-supplemented samples. The latter included biosynthesis of glycogen, cofactor-biosynthesis members (NAD, GGPP), unsaturated fatty acids biosynthesis (involving many level-5 pathways, such as fatty acid biosynthesis initiation, cis-vaccenate, palmitoleate, gondoate, oleate, and (5Z)-dodec-5-enoate biosynthesis), phospholipid biosynthesis (cdp-diacylglycerol I, II and phosphatidylglycerol I, II), purine nucleotides salvage (adenine and adenosine salvage III), and isoprenoids, together with a reduction of glycogen degradation. Notably, two pathways, biotin and stearate biosynthesis, showed a discordant behaviour in BC-supplemented samples, being increased in 2.5% BC and decreased in 5% BC compared to the CTRL diet.

### 3.6. Firmicutes:Bacteroidetes Ratio

The Firmicutes:Bacteroidetes (now so-called “Bacteroidota”, F:B) ratio was found to be very different in the mucosa and luminal samples (in both caecum and colon), with the former characterized by a higher F:B (average of 82.4 and 3.7, respectively). The F:B ratio was found to be significantly reduced in caecum lumen samples of the 2.5% BC group (*p* = 0.0112, Dunn pairwise test). On the other hand, regardless of being from the colon or mucosa, jejunum samples had a notably higher ratio (range 987.9–58117.8, no significant differences among groups) (Table 3).

## 4. Discussion

This is the first study investigating the effects of BC diet supplementation on the intestinal microbial community and its metabolism-associated pathways in rabbits. Moreover, for the first time, the differences among luminal and mucosa-associated microbiota in the jejunum, caecum, and colon of rabbits have also been investigated.

First of all, the taxonomic composition of gut microbiota independently from the experimental group was investigated. In jejunum lumen samples, about 80% of the microbial population belonged to the Firmicutes phylum. The most represented family was Eubacteriaceae, followed by Erysipelotrichaceae. This family is involved in lipid metabolism [52], confirming that it mainly occurs at the level of the jejunum [53]. On the other hand, caecum and colon luminal microbiota showed similar composition at the phyla level, with Firmicutes and Bacteroidetes as dominant phyla. They are bacteria specialized in the degradation of insoluble fibre and polysaccharide utilization [54], respectively, consistent with the fermentative functions of the caecum and colon [8,9,40]. At the family level, both Ruminococcaceae and Lachnospiraceae showed relevant abundances in the caecum and colon lumen, in agreement with other studies on rabbits [40,41]. They are responsible for the hydrolyzation of starch and other saccharides, producing short-chain fatty acids at the caecum and colon levels [55]. Regardless of the intestinal tract considered, at the genus level, the degree of microbial definition was low, probably due to the relatively poor characterization of rabbit metagenomes in the taxonomy reference database (i.e., SILVA). Among the major genera reported, *Akkermansia* and *Ruminococcus* agree with other investigations [40,41]. The mucosa-associated microbiota showed significant differences from the luminal microbiota. Firmicutes was the most abundant phylum in the jejunum and colon, while Bacteroidota prevailed in the caecum. Overall, the mucosa-associated microbiota had a less characterized microbial population with a higher percentage of undetermined taxa and, therefore, deserves further investigation.

Regardless of the experimental group, the alpha-diversity showed higher values in the caecum and colon compared to the jejunum. This was a foreseeable result, considering that the rabbit is a hindgut fermenter, and the biggest microbial population lives in the caecum and colon [8,9,40]. As regards the site, higher biodiversity was found in the luminal samples compared with the mucosal ones. In rabbits, to date, no study has compared luminal and mucosa-associated microbiota, and also in other animal species, the information is still limited and variable according to the gastrointestinal tract considered [56]. 

The analysis of the beta-diversity indicated that a different microbial composition characterizes the different tracts of the rabbit intestine, except for colon and caecum luminal samples, which showed similar results. This is probably due to the similar physiological fermentative function of these two tracts, which, therefore, have a homogenous microbial population [40]. Interestingly but not surprisingly, lumen and mucosa-associated microbiota also showed significant differences in beta-diversity, highlighting the presence of a different microbial community in these two sites. 

In this study, the effect of the BC diet supplementation on the intestinal rabbit microbiota biodiversity and microbial composition has been evaluated for the first time. The alpha-diversity index indicated several differences due to the BC administration, although only as a trend toward statistical significance. Findings suggested a dose-dependent effect of BC on the microbiota’s biodiversity of jejunum and caecum samples, but the direction of these changes (i.e., increase or decrease in diversity) was different between lumen and mucosa. In particular, the microbiota’s biodiversity in the caecum mucosa tended to increase as the dose of BC supplementation increased. Similar results were found after BC administration on mice’s gut microbiota, mainly due to an increase in short-chain fatty acids-producing microorganisms [57]. In the same investigation, the effect of BC administration was also found for the microbial composition evaluated by beta-diversity analysis. That is consistent with another investigation in mice fed a diet supplemented with goat colostrum [58] and was confirmed by our findings. Unweighted UniFrac and principal coordinates analysis showed, indeed, a different qualitative microbial composition in the three experimental groups (significant at 0.01 level). Interestingly, the 2.5% BC group greatly differed from the other groups, while the distance between CTRL and the 5% BC groups was smaller, especially in the caecum and colon. This suggests that the 2.5% BC diet supplementation can modify the microbial composition of the intestinal microbiota in rabbits and that different doses of supplementation can influence the phylogenetic microbial composition in different ways.

The relative abundance analysis showed that the greater modulation by BC mainly interested the sub-dominant components of the microbiota. Few but interesting differences were, however, found for the phyla. Specifically, Firmicutes decreased in the colon luminal samples of both BC groups, probably as a result of the reduced fibre content of BC diets. 

At the family level, the most important taxa showing significant differences among groups were Clostridia UCG-014, Barnesiellaceae, and Ruminococcaceae. Clostridia UCG-014 increased in the caecum luminal samples of BC groups. This family is positively considered as one of the main bacteria involved in the production of tryptophan metabolites, which in turn are pivotal for the regulation of gastrointestinal homeostasis in humans [59,60]. They exert a preventive effect on dysbiosis during induced ulcerative colitis, as demonstrated in mice [61], while their reduction has been highlighted in *Campylobacter jejuni*-infected turkeys [62]. Barnesiellaceae decreased in the caecum luminal samples of the 2.5% BC group. Barnesiellaceae is a less investigated family, and just a few studies have reported its increase in the gut microbiota of human patients affected by cardiovascular disease [63] and in women with a sedentary lifestyle [64]. Conversely, the Ruminococcaceae is a family which usually has a high prevalence in the rabbit gastrointestinal tract [8,9,40]. These bacteria are involved in fibre digestion and are responsible for the production of short-chain fatty acids. The present study showed a decrease in Ruminococcaceae in the colon luminal samples of the 2.5% BC group, and this could be due to the lower fibre content of the BC diets.

Other minor families were modulated by the BC administration. Specifically, Eggerthellaceae, a polyphenol-degradating family specifically correlated to lipid metabolism, has increased its prevalence in both BC groups. This could be due to a higher lipid content of the BC-supplemented diets compared to the control group. Its increment has been previously linked to weight loss and intestinal histomorphology restoration in obese mice [65]. Considering the positive role of Eggerthellaceae in gut microbiota, our result is particularly encouraging for further research in this direction. BC-supplemented rabbits also tended to have a higher abundance of Lachnospiraceae, Akkermansiaceae, and Bacteroidaceae. Lachnospiraceae are among the main producers of short-chain fatty acids in the gut microbiota, particularly butyrate, which is among the main energetic sources for colonocytes, while Akkermansiaceae metabolize the mucin present in the mucus gel layer in the colon tract. As hypothesized in another study evaluating the effect of bovine colostrum feed supplementation on mice [57], the increase of Lachnospiraceae and Akkermansiaceae could have a positive effect at multiple levels, such as on the intestinal barrier, on the nervous system development, and on the immune system of the host. Finally, the Bacteroidaceae family provides nutrients and vitamins to the organism through the metabolization of polysaccharides and oligosaccharides, and, as a consequence, it is considered beneficial for the intestinal microbiota [66]. Thus, it can be inferred that the rabbit diet supplementation with BC, especially at the 2.5% dose, could positively modulate the intestinal microbiota, even if the role and prevalence of Ruminococcaceae in the rabbit gut should be better investigated.

In this study, the gut microbiota metabolism-associated pathways were also investigated to better understand the influence that BC supplementation could have on the functional potential of the microbiota. Genes of microorganisms encode for several enzymes, which are involved in protein, lipid, carbohydrate, and nucleotide metabolism. For this reason, microbiota alterations, also due to diet modifications, are linked to gut microbiota metabolic activity variations. In our study, biosynthesis of several pathways, such as that of cofactors (e.g., quinones and vitamin B6), diterpenoids, and sugar nucleotides, was generally increased after BC supplementation. These modifications could be related to the increment of some bacterial populations, such as Eggerthellaceae, which are involved in the synthesis of some of these cofactors [67]. On the other hand, biosynthesis of amino acids (i.e., phenylalanine, tyrosine, threonine, and lysine), nucleotides, glycogen, and lipids (i.e., unsaturated fatty acids) was decreased in several tracts of BC groups. The decreased lipids biosynthesis could be related to the decrease of Firmicutes in the colon of both BC groups, as has already been noted in Grass Carp [68]. Isoprenoids, NAD, and GGPP, had contrasting results, while the amino acid degradation pathways increased in most of the intestinal tracts of the BC groups. It is worth highlighting the peculiar behavior of the amino acids’ metabolism as their biosynthesis decreases while the degradation increases. This pattern could be related to the better amino acidic profile of BC compared to feeds of plant origin [69]. It could be responsible for the higher bioavailability of amino acids at the gut level and, therefore, for the lower biosynthetic and higher degradation activity of the microbiota. Interestingly, lactose degradation was increased in the jejunum mucosa-associated microbiota of BC groups. Thus, although the lactose content in the BC diets was low, it was enough to increase its degradation pathways in microbiota. In rabbits, the lactase activity abruptly decreases after weaning and cannot increase after dietary lactose supplementation [70]. For this reason, the lactose supplemented by the diet in the weaned rabbit is not digested by the enzymatic activity of the organism and remains available for microbiota fermentations. It has been previously shown that the substitution of starch by lactose in the rabbit diet causes impaired feed efficiency and greater mortality in fattening animals caused by diarrhoea events [70]. No diarrhoea events were recorded in our study, but further investigations into the effects of colostrum on productive performance are needed.

Finally, the Firmicutes:Bacteroidetes ratio (F:B), an important index for the evaluation of the eubiosis, has also been evaluated. Its alterations have been linked to the presence of inflammatory and metabolic diseases in humans, being a good indicator of microbiota homeostasis [71]. Specifically, an increase in its value has been correlated with obesity, while its reduction has been linked to inflammatory bowel disease [72]. In rabbits, the F:B reduced after n-3 PUFA and Goji berries supplementations [8,9]. These studies also showed a progressive decrease in F:B along the gastrointestinal tract [8]. Our findings confirm this pattern, with higher F:B values in the jejunum than the caecum or colon, regardless of the experimental groups. This pattern was in agreement with the differences in the relative abundances of the microbiota of the different intestinal tracts. Our study has also found higher values of the F:B ratio in every mucosal sample when compared to the luminal ones, also reflecting, in this case, the different taxonomic compositions of the two sites. On this topic, the scientific literature is scarce, and another study on mice found opposite results [56]. As regards the diet effect, F:B only showed a decrease in the caecum luminal microbiota of the 2.5% BC group. These findings were consistent with the reduction of Ruminococcaceae and the increase of Bacteroidaceae mentioned above. In rabbits, a decrease of F:B is associated with the administration of different probiotics, although with contrasting results [8,9]. The association between F:B and metabolic disease in the rabbit remains unexplored.

Further research will be needed to better investigate the effects of the BC diet supplementation on the histological structure of the rabbit intestine, the immune response, metabolic diseases, and the animals’ productive and reproductive performances. Furthermore, it should be noted that functional predictions and the related pathways analyses performed in this study cannot substitute whole shotgun metagenomics in the evaluation of the actual functions and pathways altered by the BC-supplementation in the diet. As a matter of fact, differential abundance testing results are known to vary between shotgun metagenomics data and amplicon-based metagenome predictions based on the same samples, especially for community-wide pathway predictions [48,73]. Moreover, the short-chain fatty acids analysis could also help in the understanding of the functional modifications of the gut microbiota, together with a deeper investigation of the effect of lactose inclusion in the diet. Finally, since the percentage of BC inclusion in the diet seems to give non-linear results in microbial changes, further studies will have to establish the optimal dose of administration and its balance with the amount of fibre in the feed.

## 5. Conclusions

The results of this investigation have evidenced that dietary supplementation with BC could modulate the gut microbiota and its metabolic-associated pathways in fattening rabbits. Although microbial diversity was not strongly modified, the 2.5% BC supplementation changed the phylogenetic microbial composition, especially in the caecum and colon. Clostridia UCG-014, Barnesiellaceae, and Eggerthellaceae were the families most affected in their prevalence by the dietary treatment and altogether suggest a positive microbiota modulation exerted by the BC administration, even if the consequences of these changes should be better investigated. Further research is needed to clarify the BC dose to be added to feed and the other effects that BC inclusion could have on the rabbit besides intestinal microbial composition changes.

## Figures and Tables

**Table 1:** Analytical chemical composition of control (CTRL) and experimental diets supplemented with 2.5% (2.5% BC) and 5% (5% BC) of bovine colostrum.

Chemical Composition	Group		
	CTRL	2.5% BC	5% BC
Dry matter	92.34	91.71	91.69
Crude protein	14.82	14.76	15.23
Ether extract	2.79	2.95	3.02
Ash	7.04	7.23	7.62
NDF ^1^	40.00	36.81	35.79
ADF ^2^	27.04	24.92	24.31
ADL ^3^	12.02	10.03	9.11

^1^ NDF: neutral detergent fibre; ^2^ ADF: acid detergent fibre; ^3^ ADL: acid detergent lignin.

**Table 2:** Table of level-4 MetaCyc pathways found as differentially abundant (*p* < 0.05) in samples from rabbits fed a BC-supplemented diet, separated on intestinal tracts and sampling sites. For each level-4 pathway, the corresponding upper lineage classification is reported. “BS”: “biosynthesis”; “DEG”: “Degradation”. The arrow direction indicates whether the pathway was enriched (more abundant) or depleted (less abundant) in BC-supplemented samples (both for 2.5% BC and 5% BC). ↔ indicates that there was a contrasting behaviour for 2.5% BC and 5% BC samples with respect to the CTRL diet.

L2/L3 Pathway	L4 Pathway	Jejunum		Caecum		Colon	
		Lumen	Mucosa	Lumen	Mucosa	Lumen	Mucosa
Cofactor biosynthesis	NAD-BS	↓	↑		↓		
	GGPP-BS		↑			↓	↓
	Biotine-BS						↔
	DHNA-BS					↑	
	demethylmenaquinone-BS					↑	
	menaquinone-BS					↑	
	phylloquinone-BS	↑				↑	
	vitamin B6-BS			↑		↑	
Terpenoids biosynthesis	diterpenoids-BS		↑				
	isoprenoids-BS		↑			↓	↓
Amino acid biosynthesis	phenylalanine-BS	↓		↔			
	tyrosine-BS	↓		↔			
	threonine-BS			↓			
	lysine-BS			↓			
Amino acid degradation	Lysine-DEG		↑	↑			↑
	Histidine-DEG			↑	↑		↑
	Glutamate-DEG					↑	
Nucleotide biosynthesis	5-aminoimidazole ribonucleotide-BS			↓			
	purine nucleotides salvage			↓		↓	↓
Carbohydrate biosynthesis	Glycogen-BS					↓	↓
	sugar nucleotides-BS			↑			
Carbohydrate degradation	lactose-DEG		↑				
Lipid biosynthesis	unsaturated fatty acids-BS						↓
	cdp-diacylglycerol-BS						↓
	phosphatidylglycerol-BS						↓
	stearate-BS						↔

**Table 3:** Average Firmicutes:Bacteroidetes ratio for the three diet groups (CTRL, 2.5% BC, and 5% BC) divided per intestinal tract and tissue. The *p*-value is that of a Kruskal–Wallis test; “*” indicates statistical significance (*p* < 0.05). In Diet groups, a significant difference from the CTRL diet (Dunn’s post hoc pairwise test, *p* < 0.05) is indicated by a “§”.

Intestinal Tract	Site	Diet Group			*p*-Value
		CTRL	2.5% BC	5% BC	
Jejunum	Lumen	57,981.4	29,803.4	58,117.8	0.396
	Mucosa	29,271.3	15,290.1	987.9	0.762
Caecum	Lumen	3.8	3.1 ^§^	4.2	0.046 *
	Mucosa	72.7	15.5	354.3	0.224
Colon	Lumen	4.4	3.5	3.4	0.212
	Mucosa	16.8	14.7	20.6	0.478

## Data Availability

The data presented in this study are openly available in NCBI Short Read Archive (SRA) under experiment IDs SRR22879769-SRR22879893 (BioProject ID PRJNA915237, https://www.ncbi.nlm.nih.gov/bioproject/PRJNA915237, accessed on 5 January 2023).

## Supplementary Materials

The following supporting information can be downloaded at: https://www.mdpi.com/article/10.3390/ani13060976/s1, Figure S1: Rarefaction curves of the alpha-diversity metrics (chao1 and observed species) for each of the 125 samples; Figure S2: Barplots representing the relative abundance composition of the 125 samples at all taxonomic levels; Figure S3: Boxplots of the alpha-diversity divided according to (A) intestinal tract and site, (C) intestinal tract, and (E) site of the samples; Principal Coordinate Analysis (PCoA) based on the weighted UniFrac distances, colored according to (B) intestinal tract and site, (C) intestinal tract and (D) site; Table S1: *p*-values from the permutation-based non-parametric tests on alpha-diversity estimations (PD whole tree metrics) for samples divided by intestinal tract and site; Table S2: *p*-values from the “Adonis”-based tests on beta-diversity distances (unweighted and weighted UniFrac) for samples divided by intestinal tract and site; Table S3: *p*-values from the permutation-based non-parametric tests on alpha-diversity estimations (PD whole tree metrics) for samples divided by intestinal tract; Table S4: *p*-values from the “Adonis”-based tests on beta-diversity distances (unweighted and weighted UniFrac) for samples divided by intestinal tract; Table S5: *p*-values from the “Adonis”-based tests on beta-diversity distances (unweighted UniFrac) stratifying samples in diet groups homogeneous for intestinal tract and site sampled; Table S6: *p*-values from the “Adonis”-based tests on beta-diversity distances (unweighted UniFrac) for samples divided by BC supplementation in diet; Table S7: Table reporting in full the evidences from the relative abundance analysis, per intestinal tract and site, at all the phylogenetic levels.
