# Supplementary material for: Bovine Colostrum Supplementation Modulates the Intestinal Microbial Community in Rabbits
Source: Animals (Basel). 2023 Mar 8;13(6):976. doi: 10.3390/ani13060976 (PMC10044174; doi:10.3390/ani13060976)
Supplement: Supplementary file 1 [file animals-13-00976-s001.zip › animals-2182380-supplementary.pdf]

Supplementary material

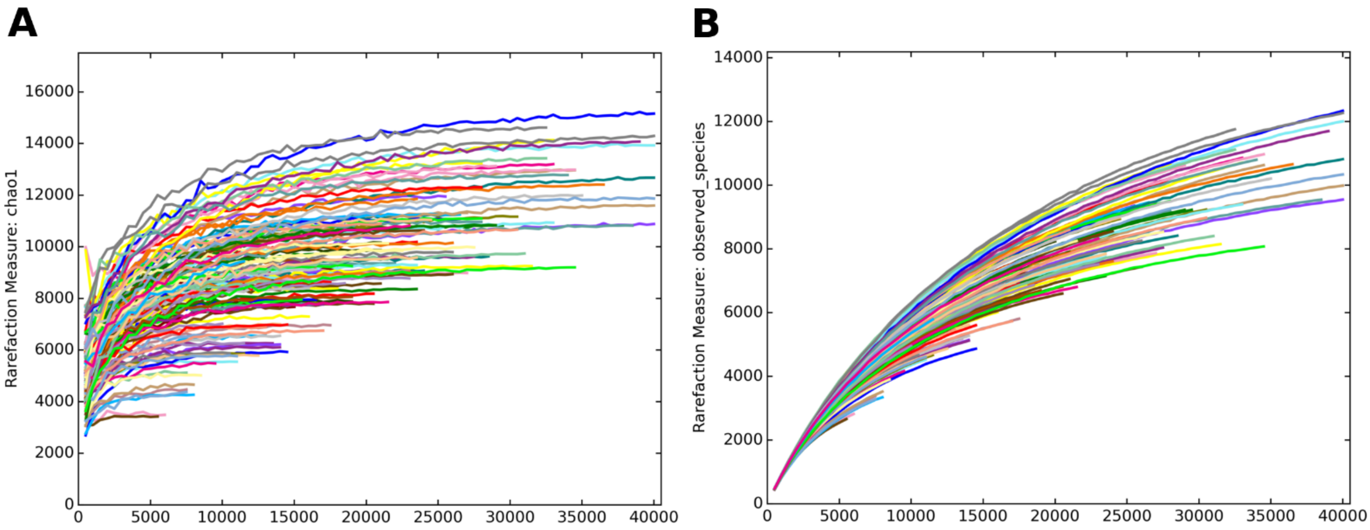

Figure S1. Rarefaction curves of the alpha-diversity metrics (chao1 and observed species) for each of the 125 samples. Curves were obtained rarefying the number of sequences per sample in steps of 500 sequences each.

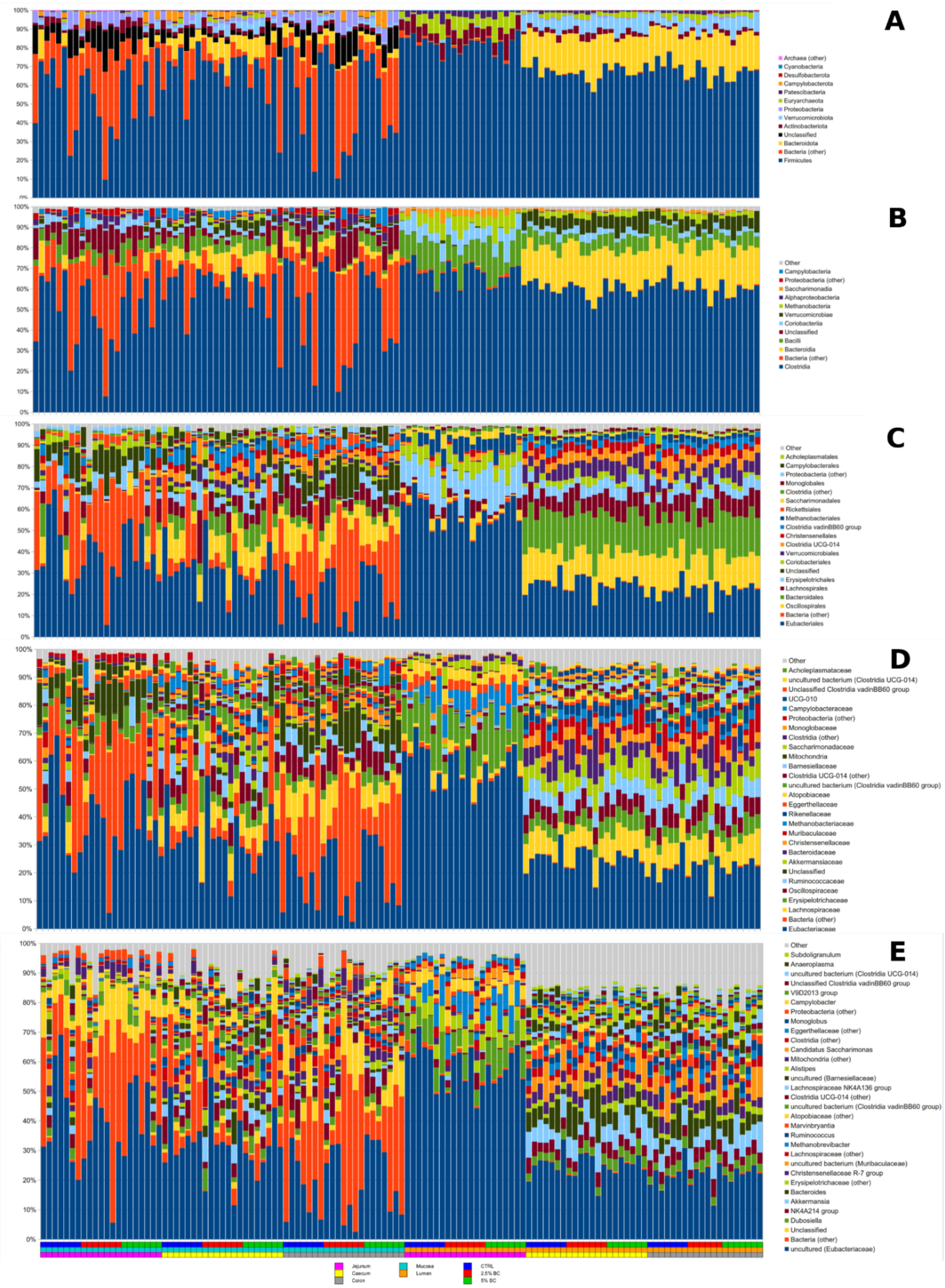

Figure S2. Barplots representing the relative abundance composition of the 125 samples at all taxonomic levels. Taxa with average abundance over the samples <1% were grouped in the "Other" category.

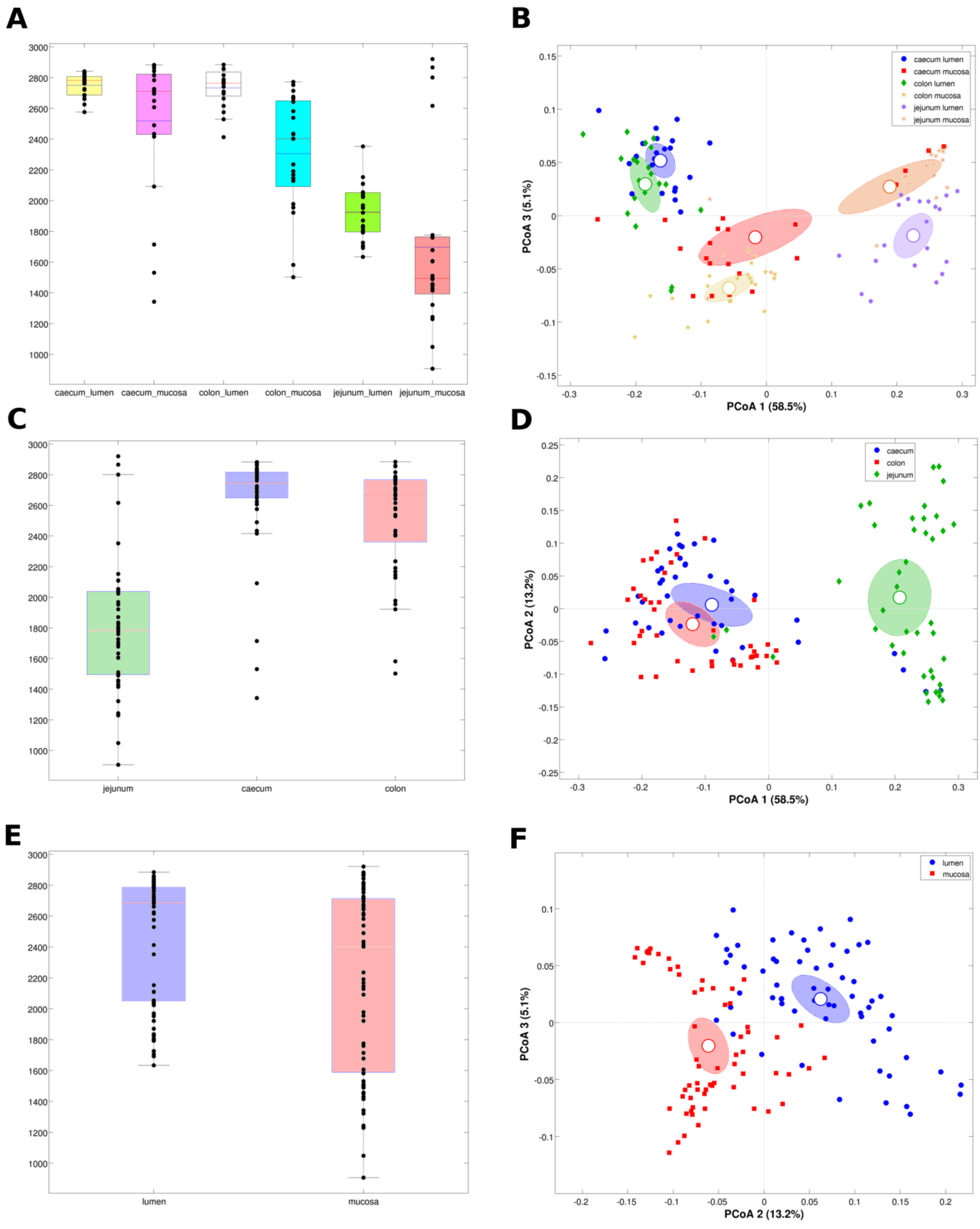

Figure S3. (A, C, E) Boxplots of the alpha-diversity (PD whole tree metric) divided according to (A) intestinal tract and site, (C) intestinal tract, and (E) site of the samples. Red line represents the median values, whereas blue lines represent the mean values; each point represents a sample. (B, D, F) Principal Coordinate Analysis (PCoA) based on the weighted UniFrac distances; in each plot, each dot represents a sample, colored according to (B) intestinal tract and site, (D) intestinal tract and (F) site. Ellipses are the 95% SEM-based confidence intervals, whereas centroids are the average coordinates on all the samples.

Table S1. P-values from the permutation-based non-parametric tests on alpha-diversity estimations (PD whole tree metrics) for samples divided by intestinal tract and site. Only significant (p<0.05) p-values are reported. "." Indicates a non-significant difference.

	Jejunum lumen	Jejunum mucosa	Caecum lumen	Caecum mucosa	Colon lumen	Colon mucosa
Jejunum lumen	--	.	0.015	0.015	0.015	0.015
Jejunum mucosa	.	--	0.015	0.015	0.015	0.015
Caecum lumen	0.015	0.015	--	.	.	0.015
Caecum mucosa	0.015	0.015	.	--	.	.
Colon lumen	0.015	0.015	.	.	--	0.015
Colon mucosa	0.015	0.015	0.015	.	0.015	--

Table S2. P-values from the "Adonis"-based tests on beta-diversity distances (unweighted and weighted UniFrac) for samples divided by intestinal tract and site. Only significant (p<0.05) p-values are reported. "." Indicates a non-significant difference.

		Jejunum lumen	Jejunum mucosa	Caecum lumen	Caecum mucosa	Colon lumen	Colon mucosa
Unweighted UniFrac	Jejunum lumen	--	0.001	0.001	0.001	0.001	0.001
	Jejunum mucosa	0.001	--	0.001	0.001	0.001	0.001
	Caecum lumen	0.001	0.001	--	0.001	.	0.001
	Caecum mucosa	0.001	0.001	0.001	--	0.001	0.001
	Colon lumen	0.001	0.001	.	0.001	--	0.001
	Colon mucosa	0.001	0.001	0.001	0.002	0.001	--
Weighted UniFrac	Jejunum lumen	--	0.001	0.001	0.001	0.001	0.001
	Jejunum mucosa	0.001	--	0.001	0.001	0.001	0.001
	Caecum lumen	0.001	0.001	--	0.001	.	0.001
	Caecum mucosa	0.001	0.001	0.001	--	0.001	0.021
	Colon lumen	0.001	0.001	.	0.001	--	0.001
	Colon mucosa	0.001	0.001	0.001	0.02	0.001	--

Table S3. P-values from the permutation-based non-parametric tests on alpha-diversity estimations (PD whole tree metrics) for samples divided by intestinal tract. Only significant (p<0.05) p-values are reported. "." Indicates a non-significant difference.

	Jejunum	Caecum	Colon
Jejunum	--	0.003	0.003
Caecum	0.003	--	.
Colon	0.003	.	--

Table S4. P-values from the "Adonis"-based tests on beta-diversity distances (unweighted and weighted UniFrac) for samples divided by intestinal tract. Only significant (p<0.05) p-values are reported. "." Indicates a non-significant difference.

		Jejunum	Caecum	Colon
Unweighted UniFrac	Jejunum	--	0.001	0.001
	Caecum	0.001	--	0.001
	Colon	0.001	0.001	--
Weighted UniFrac	Jejunum	--	0.001	0.001
	Caecum	0.001	--	0.021
	Colon	0.001	0.02	--

Table S5. P-values from the “Adonis”-based tests on beta-diversity distances (unweighted UniFrac) stratifying samples in diet groups homogeneous for intestinal tract and site sampled (n=7), i.e., considering differences only on the diet supplementation provided the intestinal tract (jaejunum, caecum, or colon) and site (lumen and mucosa) were the same. Only significant (p<0.05) p-values are reported. “.” Indicates a non-significant difference.

		Tissue	Caecum						Colon						Jejunum					
		Site	Lumen			Mucosa			Lumen			Mucosa			Lumen			Mucosa		
Tissue	Site	Group	CTRL	2.5%(A)	5%(B)	CTRL	2.5%(A)	5%(B)	CTRL	2.5%(A)	5%(B)	CTRL	2.5%(A)	5%(B)	CTRL	2.5%(A)	5%(B)	CTRL	2.5%(A)	5%(B)
Jejunum	Lumen	CTRL	0.003	0.002	0.001	0.004	0.003	0.001	0.003	0.002	0.001	0.002	0.002	0.001	--	.	.	.	0.002	0.002
		2.5%(A)	0.002	0.002	0.002	0.005	0.003	0.002	0.001	0.001	0.002	0.001	0.001	0.003	.	--	.	.	0.014	0.012
		5%(B)	0.001	0.001	0.002	0.002	0.001	0.006	0.005	0.003	0.002	0.001	0.002	0.002	.	.	--	.	0.001	0.011
	Mucosa	CTRL	0.012	0.002	0.001	.	0.003	.	0.004	0.005	0.001	0.007	0.007	0.006	.	.	.	--	.	.
		2.5%(A)	0.001	0.001	0.002	0.017	0.001	0.007	0.002	0.002	0.001	0.002	0.001	0.001	0.003	0.016	0.001	.	--	.
		5%(B)	0.001	0.002	0.002	0.019	0.003	0.017	0.001	0.004	0.002	0.002	0.005	0.004	0.003	0.007	0.005	.	.	--
Caecum	Lumen	CTRL	--	0.002	0.001	.	0.001	0.002	.	0.001	0.001	0.002	0.002	0.002	0.001	0.001	0.002	0.017	0.002	0.002
		2.5%(A)	0.001	--	0.002	0.001	0.036	0.003	0.001	.	0.001	0.002	0.001	0.002	0.002	0.001	0.001	0.002	0.004	0.002
		5%(B)	0.001	0.002	--	0.003	0.001	.	0.001	0.001	.	0.001	0.001	0.004	0.002	0.002	0.002	0.002	0.002	0.002
	Mucosa	CTRL	.	0.001	0.001	--	.	.	0.001	0.001	0.001	.	0.001	0.001	0.001	0.001	0.001	.	0.012	0.027
		2.5%(A)	0.001	0.042	0.001	.	--	.	0.003	0.04	0.001	0.001	.	0.001	0.001	0.001	0.002	0.008	0.005	0.002
		5%(B)	0.001	0.001	.	.	.	--	0.001	0.002	.	0.008	0.01	.	0.007	0.007	0.007	.	0.014	0.032
Colon	Lumen	CTRL	.	0.001	0.002	0.016	0.003	0.002	--	0.001	0.001	0.014	0.002	0.002	0.003	0.001	0.003	0.01	0.002	0.001
		2.5%(A)	0.001	.	0.001	0.002	0.026	0.003	0.003	--	0.002	0.002	0.002	0.013	0.004	0.002	0.002	0.011	0.001	0.001
		5%(B)	0.001	0.005	.	0.001	0.002	.	0.003	0.001	--	0.002	0.001	0.011	0.002	0.001	0.002	0.003	0.001	0.002
	Mucosa	CTRL	0.006	0.001	0.001	.	0.001	0.005	0.016	0.004	0.001	--	.	0.016	0.003	0.001	0.001	0.006	0.001	0.002
		2.5%(A)	0.001	0.003	0.003	0.004	.	0.008	0.001	0.001	0.019	.	--	0.018	0.002	0.004	0.001	0.002	0.002	0.001
		5%(B)	0.002	0.002	0.002	0.002	0.001	.	0.002	0.001	0.008	0.017	0.019	--	0.001	0.001	0.002	0.003	0.002	0.004

Table S6. P-values from the "Adonis"-based tests on beta-diversity distances (unweighted UniFrac) for samples divided by BC supplementation in diet. Only significant ($p<0.05$) p-values are reported. "." Indicates a non-significant difference.

	CTRL	2.5%(A)	5%(B)
CTRL	--	0.003	0.014
2.5%(A)	0.007	--	0.006
5%(B)	0.007	0.003	--

Table S7. Table reporting in full the evidences from the relative abundance analysis, per intestinal tract and site, at all the phylogenetic levels. Taxa underlined are those significantly (p<0.05, Kruskal-Wallis test) altered by the colostrum addition to diet. The others are those showing a tendency of an increase/reduction. In both, CTRL diet was considered as a reference.

		**Jejunum**		**Caecum**		**Colon**	
Phylum	**Lumen**	--	--	Desulfobacterota<br>Firmicutes<br>Bacteroidota<br>Verrucomicrobiota<br>Euryarchaeota	Increased in 2.5%<br>Decreased in 2.5%<br>Increased in 2.5%<br>Increased in 5%<br>Increased in 5%	Firmicutes<br>Bacteroidota<br>Actinobacteriota	Decreased in 2.5% and 5%<br>Increased in 2.5% and 5%<br>Increased in 2.5% and 5%
	**Mucosa**	Firmicutes<br>Bacteroidota	Decreased in 2.5%<br>Decreased in 2.5% and 5%	Bacteroidota<br>Verrucomicrobiota<br>Euryarchaeota	Increased in 2.5% and 5%<br>Increased in 2.5% and 5%<br>Increased in 5%	Firmicutes<br>Campylobacterota	Decreased in 2.5%<br>Increased in 2.5% and 5%
Class	**Lumen**	Clostridia<br>Bacilli	Decreased in 5%<br>Decreased in 2.5%	Desulfovibrionia<br>Negativicutes<br>Clostridia<br>Bacteoroidia	Increased in 2.5%<br>Increased in 2.5% and 5%<br>Decreased in 2.5%<br>Increased in 2.5%	Negativicutes<br>Clostridia<br>Verrucomicrobiae	Increased in 2.5% and 5%<br>Decreased in 2.5% and 5%<br>Increased in 5%
	**Mucosa**	Clostridia<br>Bacteria (other)<br>Bacteroidia	Decreased in 2.5%<br>Increased in 2.5%<br>Decreased in 2.5% and 5%	Negativicutes<br>Clostridia<br>Bacteoroidia<br>Verrucomicrobiae<br>Campylobacteria	Increased in 2.5%<br>Increased in 2.5%<br>Increased in 2.5% and 5%<br>Increased in 2.5% and 5%<br>Decreased in 5%	Negativicutes<br>Clostridia<br>Campylobacteria	Increased in 2.5% and 5%<br>Decreased in 2.5%<br>Increased in 2.5% and 5%
Order	**Lumen**	Clostridia (other)	Increased in 2.5% and 5%	Erysipelotrichales<br>Clostridia (vadinBB60)<br>Bacteroidales	Decreased in 5%<br>Increased in 2.5% and 5%<br>Increased in 2.5%	Monoglobales	Increased in 2.5% and 5%
	**Mucosa**	Eubacteriales<br>Bacteria (other)<br>Oscillospirales<br>Bacteroidales<br>Lachnospirales	Decreased in 2.5%<br>Increased in 2.5%<br>Decreased in 2.5%<br>Decreased in 2.5% and 5%	Clostridia (other)<br>Eubacteriales<br>Bacteroidales<br>Lachnospirales<br>Verrucomicrobiales	Increased in 2.5%<br>Decreased in 2.5% and 5%<br>Increased in 2.5% and 5%<br>Increased in 2.5%	Eubacteriales<br>Bacteria (other)<br>Campylobacterales	Increased in 5%<br>Decreased in 2.5% and 5%<br>Increased in 2.5% and 5%

		Clostridia (vadinBB60)<br>Clostridiales	Decreased in 2.5% and 5%<br>Decreased in 2.5% and 5%<br>Increased in 2.5%	Campylobacterales	Increased in 2.5% and 5%<br>Decreased in 5%		
Family	**Lumen**	Clostridia (other)<br>Eubacteriaceae<br>Erysipelotrichaceae<br>Oscillospiraceae<br>Ruminococcaceae	Increased in 2.5% and 5%<br>Decreased in 2.5%<br>Decreased in 2.5%<br>Increased in 2.5%<br>Increased in 2.5%	Erysipelotrichaceae<br>Eggerthellaceae<br>Clostridia UCG-014<br>Barnesiellaceae<br>UCG-010<br>Clostridia (vadinBB60)<br>Bacteroidaceae	Increased in 5%<br>Increased in 2.5% and 5%<br>Increased in 2.5% and 5%<br>Decreased in 2.5%<br>Decreased in 5%<br>Decreased in 5%<br>Increased in 2.5%	Ruminococcaceae<br>Eggerthellaceae<br>Monoglobaceae<br>Akkermansiaceae<br>Bacteroidaceae<br>Rikenellaceae	Decreased in 2.5%<br>Increased in 2.5% and 5%<br>Decreased in 2.5% and 5%<br>Increased in 5%<br>Increased in 2.5%<br>Increased in 5%
	**Mucosa**	Eubacteriaceae<br>Bacteria (other)<br>Lachnospiraceae<br>Clostridiaceae	Decreased in 2.5%<br>Increased in 2.5%<br>Decreased in 2.5% and 5%<br>Increased in 2.5%	Clostridia (other)<br>Eubacteriaceae<br>Bacteria (other)<br>Lachnospiraceae<br>Akkermansiaceae<br>Bacteroidaceae<br>Barnesiellaceae<br>Campylobacteraceae	Increased in 2.5%<br>Decreased in 5%<br>Decreased in 2.5%<br>Increased in 2.5%<br>Increased in 2.5% and 5%<br>Increased in 2.5%<br>Increased in 5%<br>Decreased in 5%	Eubacteriaceae<br>Bacteria (other)<br>Campylobacteraceae	Increased in 5%<br>Decreased in 5%<br>Increased in 2.5% and 5%
Genus	**Lumen**	Lachnospiraceae (NK4A136)<br>Unc. Clostridia<br>Unc. Eubacteriaceae<br>Dubosiella<br>Christensenellaceae (R-7)	Decreased in 2.5%<br>Increased in 2.5% and 5%<br>Decreased in 2.5%<br>Decreased in 2.5%<br>Increased in 2.5%	Dubosiella<br>Unc. Erysipelotrichaceae<br>Clostridia (vadinBB60)<br>Clostridia (UCG-014)<br>Lachnospiraceae (NK4A136)<br>Unc. Barnesiellaceae<br>Unc. Eggerthellaceae<br>Unc. UCG-010<br>Eubacterium (siraeum)<br>Bacteroides	Increased in 5%<br>Increased in 5%<br>Decreased in 5%<br>Increased in 5%<br>Increased in 5%<br>Decreased in 2.5%<br>Increased in 5%<br>Decreased in 5%<br>Increased in 2.5% and 5%<br>Increased in 2.5%	Unc. Eggerthellaceae<br>Monoglobus<br>Akkermansia<br>Bacteroides<br>Anaeroplasma	Increased in 5%<br>Decreased in 2.5% and 5% Increased in 5%<br>Increased in 2.5%<br>Decreased in 2.5% and 5%
	**Mucosa**	Unc. Eubacteriaceae<br>Bacteria (other)<br>Unclassified	Decreased in 2.5%<br>Increased in 2.5%<br>Increased in 2.5%	Clostridia (other)<br>Bacteria (other)<br>Akkermansia	Increased in 2.5%<br>Decreased in 2.5%<br>Increased in 2.5% and 5%	Unc. Eubacteriaceae<br>Bacteria (other)<br>Campylobacter	Increased in 5%<br>Decreased in 5%<br>Increased in 2.5% and 5%

		NK4A214 group<br>Unc. Atopobiaceae<br>Clostridia (vadinBB60)<br>Candidatus Arthromitus	Decreased in 2.5% and 5%<br>Increased in 2.5% and 5%<br>Decreased in 2.5% and 5%<br>Increased in 2.5%	Bacteroides<br>Unc. Lachnospiraceae<br>Marvinbryantia<br>Campylobacter	Increased in 2.5% and 5%<br>Increased in 2.5% and 5%<br>Increased in 2.5%<br>Decreased in 5%	V9D2013 group<br>Clostridia (vadinBB60)<br>Anaeroplasma	Increased in 5%<br>Decreased in 2.5% and 5%<br>Decreased in 2.5% and 5%
